# Lipid Metabolism Was Associated With Oocyte *in vitro* Maturation in Women With Polycystic Ovarian Syndrome Undergoing Unstimulated Natural Cycle

**DOI:** 10.3389/fcell.2021.719173

**Published:** 2021-09-03

**Authors:** Tao Liu, Dongming Liu, Xueling Song, Jiangxue Qu, Xiaoying Zheng, Jia Li, Rui Yang, Shuo Yang, Xi Zhang, Haiyan Wang, Liying Yan, Caihong Ma, Rong Li, Jie Yan, Jie Qiao

**Affiliations:** ^1^Center for Reproductive Medicine, Department of Obstetrics and Gynecology, Peking University Third Hospital, Beijing, China; ^2^National Clinical Research Center for Obstetrics and Gynecology, Beijing, China; ^3^Key Laboratory of Assisted Reproduction, Ministry of Education, Beijing, China; ^4^Beijing Key Laboratory of Reproductive Endocrinology and Assisted Reproduction, Beijing, China; ^5^Research Units of Comprehensive Diagnosis and Treatment of Oocyte Maturation Arrest, Chinese Academy of Medical Sciences, Beijing, China

**Keywords:** unstimulated natural cycle, lipid metabolism, number of oocyte retrieved, oocyte quality, clinical outcomes, polycystic ovarian syndrome

## Abstract

**Objective:**

Hyperlipidemia are common polycystic ovarian syndrome (PCOS)-related metabolic dysfunctions and can adversely affect assisted reproductive technology (ART) outcomes in controlled ovarian hyperstimulation (COH) cycles. The objective of this study is to analyze the relationship between lipid metabolism and ART outcomes in unstimulated natural cycles without the utilization of ovarian induction drugs, which is still uncertain.

**Methods:**

This retrospective study included infertile women with PCOS between 21 and 40 years old undergoing unstimulated natural cycles from January 01, 2006 to December 31, 2019. Lipid metabolism was measured by body mass index (BMI) and serum biochemical parameters including total cholesterol (TC), triglycerides (TG), high and low density lipoprotein cholesterol (HDL-C and LDL-C). ART outcomes were measured by number of oocytes retrieved, oocyte maturation quality and developmental potential, clinical pregnancy and live birth.

**Results:**

A total of 586 patients were included in this study. Multivariate Poisson log-linear analysis showed that high TC (≥5.18 mmol/L), triglycerides (TG) (≥1.76 mmol/L), LDL-C (≥3.37 mmol/L) levelsand low HDL-C levels (≤1.04 mmol/L) were significantly (*P*_*TC*_ = 0.001, *P_*TG*_* < 0.001, *P_*HDL*__–C_* < 0.001, *P_*LDL*__–C_* < 0.001) associated with increased number of oocytes retrieved. BMI was significantly negatively associated with maturation rate (*P* < 0.001), fertilization rate (*P* < 0.001) and transferrable embryo rate (*P* = 0.002). High TG levels and low HDL-C levels were also associated with decreased maturation rate (*P_*TG*_* < 0.001, *P*_*HDL–C*_ = 0.026). Logistic regression analysis showed statistically significant association between obesity (≥28.0 kg/m^2^) and decreased live birth rate (*P* = 0.004) as well as cumulative live birth rate (*P* = 0.007).

**Conclusion:**

This is the first study that focused on the relationship between basal lipid metabolism and ART outcomes in women with PCOS undergoing unstimulated natural cycles. The results showed that high levels of lipid metabolic parameters were associated with increased number of oocytes retrieved and obesity was closely associated with impaired oocyte maturation quality and developmental potential as well as poor live birth outcomes.

## Introduction

Polycystic ovary syndrome (PCOS) is a common endocrinal disorder in women at reproductive age and accounts for nearly 80% of women with anovulatory infertility (Thessaloniki ESHRE/ASRM-Sponsored PCOS Consensus Workshop Group, 2008). PCOS is characterized as anovulation, hyperandrogenism and polycystic ovary and is commonly associated with high risk of metabolic syndrome including insulin resistance, obesity, hyperlipidaemia and hypertension (De Sousa and Norman, 2016) which exacerbate the infertility of these women (Qiao and Feng, 2011).

Assisted reproductive therapies for infertile women with PCOS are commonly divided into two types depending on whether or not ovarian induction drugs are applied: controlled ovarian hyperstimulation (COH) treatment and natural cycle *in vitro* maturation (IVM) treatment, in which COH treatment is the most recommended (Balen et al., 2016). Lipid metabolic disorders including obesity and hyperlipidaemia are common PCOS-related metabolic dysfunctions, and are proven to adversely affect assisted reproductive technology (ART) outcomes of COH and human chorionic gonadotropin (hCG)-primed natural cycles in previous studies (Shalom-Paz et al., 2011; Moragianni et al., 2012; Bailey et al., 2014; Kudesia et al., 2018; Zarezadeh et al., 2020). Maternal obesity also has some adverse pregnancy consequences for both mother and child such as gestational hypertension, spontaneous pregnancy loss, premature delivery and congenital anomalies (Catalano and Shankar, 2017). However, the association between lipid metabolism and ART outcomes in unstimulated natural cycles has never been discussed before.

The main difference between COH cycle and unstimulated natural cycle is the use of ovarian induction drugs, which may affect individual’s lipid metabolism (Dhanju et al., 2001; Wang et al., 2015). Previous studies showed that gonadotropin could increase the expressions of genes related to cholesterol and steroid synthesis, such as 3-hydroxy-3-methylglutaryl-coenzyme A reductase, low density lipoprotein receptor (*Ldlr*), scavenger receptor class B member 1 (*Scarb1*) and luteinizing hormone receptor (*Lhr*) in mice (Wang et al., 2015), making the lipid profile different from the basal status before gonadotropin’s application and thus probably covering up the direct effect of basal lipid metabolism on reproductive potential. Therefore, It is uncertain whether or not the adverse effect of obesity on ART outcomes is direct due to the influence of ovarian induction drugs, the external factor in COH cycles, on lipid metabolism and ART outcomes. To clarify this question, it is essential to study the possible relationship between basal lipid metabolism and ART outcomes by ruling out the influence of ovarian induction drugs in women with PCOS.

This study included women with PCOS undergoing unstimulated natural cycles without any ovarian induction drugs applied before oocyte retrieval. The aim is to explore the relationship between basal lipid metabolism and ART outcomes including number of oocytes retrieved, oocyte maturation and embryo development quality, and clinical outcomes in women with PCOS undergoing unstimulated natural cycles, thus figure out the direct effect of lipid metabolism on reproductive potential of PCOS.

## Materials and Methods

### Patients

This retrospective study included 586 infertile women with PCOS between 21 and 40 years old undergoing unstimulated natural cycles at the Reproductive Medicine Center of Peking University Third Hospital from January 01, 2006 to December 31, 2019 inclusion criteria were as follows: (i) patients diagnosed with PCOS based on Rotterdam Criteria (Rotterdam ESHRE/ASRM-Sponsored PCOS Consensus Workshop Group, 2004), (ii) patients undergoing natural cycle treatment. Exclusion criteria were as follows: (i) patients undergoing modified natural cycle with recombinant follicle stimulating hormone (rFSH)/human chorionic gonadotropin (hCG) triggering, (ii) one or both of the couples had chromosomal abnormality, (iii) patients with thyroid dysfunction, (iv) patients with FSH, luteinizing hormone (LH) abnormalities or hyperandrogenism caused by other factors. The study had got approval from the institutional review board of Peking University Third Hospital and was approved by the Ethics Committee of Peking University (2018S2-007).

### Oocyte Retrieval and IVM Protocol

The protocol for unstimulated natural cycles was as follows. No ovulation induction treatment was used before oocyte retrieval. Oocyte retrieval was performed when antral follicles with 8–10 mm were seen in the ovary by ultrasound. Immature cumulus-oocyte complexes (COCs) were obtained through immature follicle aspiration under the transvaginal ultrasound guidance and transferred into *in vitro* fertilization (IVF) laboratory immediately.

Cumulus-oocyte complexes from follicular fluid were cultured in IVM media (IVM media kit, Origio, Denmark) with 0.075IU/mL FSH and LH (Menopur, Ferring, Germany) added at 37°C in 5% CO_2_ atmosphere for 24–48 h to maturation and then assessed for maturity according to the presence of germinal vesicle (GV) and the first polar body. The mature oocytes obtained were either frozen by vitrification for fertility preservation or fertilized by intra-cytoplasmic sperm injection (ICSI). The injected oocytes were cultured in G-1 plus media (Vitrolife, Sweden) for 3 days and sequentially in G-2 plus media (Vitrolife, Sweden) for 3–4 days. The presence of two pronuclei (2PN) and two polar bodies was checked in 16–18 h after ICSI. The embryos developmental status was assessed and graded according to previous methods (Racowsky et al., 2010) in the third, fifth and sixth day. Embryo quality was assessed according to the number, size and fragmentation of blastomeres (Zheng et al., 2012). The obtained blastula was freshly transferred or frozen for late frozen embryo transfer.

One or two best quality blastocysts were selected for fresh embryo transfer using sterile embryo transfer catheter (K-Soft 5100, Cook, Australia) and the others were cryopreserved by vitrification.

### Endometrium Preparation and Embryo Transfer

Patients receiving fresh embryo transfer were given oral estradiol valerate (6 mg daily, Progynova, Bayer, Germany) from the day of oocyte retrieval and intramuscular progesterone (60 mg daily, Xianju Pharmaceutical Trading Company, China) injection for luteal phase support from the day of ICSI. The endometrium preparation and luteal phase support were continued until a negative pregnancy test or discernible heartbeats by ultrasound.

The artificial endometrium preparation was adopted for patients receiving frozen embryo transfer. Patients were given estradiol valerate (6 mg daily) from the second day of menstruation for at least 12 days. Transvaginal ultrasound was performed after 10 days of medication to monitor the endometrial thickness. When the endometrial thickness was ≥8 mm, 8% progesterone vaginal gel (90 mg daily, Crinone, Merck, America) and oral dydrogesterone was given for luteal phase support. Frozen-thawed embryos were transferred after 7 days of luteal phase support.

### Study Design

**FIGURE 1:**
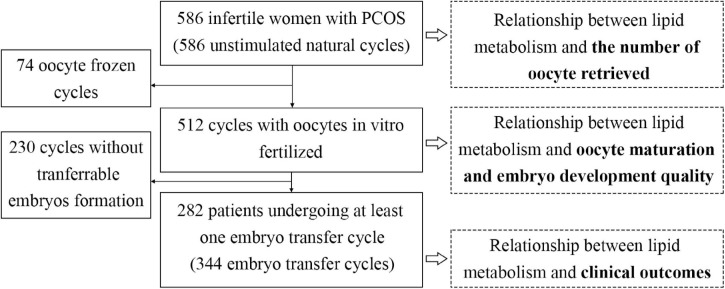
Flowchart of the analysis.

This study was designed to examine the relationship between lipid metabolism and (i) the number of oocytes retrieved, (ii) oocyte maturation quality and developmental potential, and (iii) clinical outcomes of embryo transfer cycle (Figure 1). Lipid metabolic levels were measured by body mass index (BMI) and serum biochemical parameters including total cholesterol (TC), triglycerides (TG), high and low density lipoprotein cholesterol (HDL-C and LDL-C) tested within 3 months before natural cycles start date. Patients were divided into 2–3 groups according to their BMI (<24, 24–27.9, and ≥28 kg/m^2^) (Chen, 2008), TC (<5.18 and ≥5.18 mmol/L), TG (<1.76 and ≥1.76 mmol/L), HDL-C (≤1.04 and >1.04 mmol/L), and LDL-C (<3.37 and ≥3.37 mmol/L) (Joint committee issued Chinese guideline for the management of dyslipidemia in adults, 2016).

Maturation rate per oocyte (MR, the number of oocyte in MII/the number of oocytes retrieved), fertilization rate per oocyte (FR, the number of zygote with 2 pronuclei/the number of oocytes retrieved) and transferrable embryos rate per oocyte (TER, the number of transferrable embryos/the number of oocytes retrieved) were calculated to measure the quality and developmental potential of oocytes retrieved in unstimulated natural cycles.

Clinical pregnancy was defined as the ultrasound visualization of at least one gestational sac on 30 days after ET. Live birth was defined as the delivery of at least one newborn, irrespective of the gestation (Song et al., 2020). Clinical outcomes of embryo transfer cycles were measured by clinical pregnancy rate per cycle (CPR, the number of cycles achieving clinical pregnancy/the number of embryo transfer cycles), cumulative clinical pregnancy rate (CCPR, the number of patients achieving clinical pregnancy/the number of patients undergoing at least one embryo transfer cycle), live birth rate per cycle (LBR, the number of cycles achieving live birth/the number of embryo transfer cycles), cumulative live birth rate (CLBR, the number of patients achieving live birth/the number of patients undergoing at least one embryo transfer cycle).

In addition, FSH, LH and testosterone (T) were tested between the second and fifth day of menstruation. Previous ovarian surgery included oophorocystectomy, ovarian drilling surgery and ovarian tumor surgery. Ovarian disorders other than PCOS included ovarian cyst, endometrial cyst and teratoma. Antral follicle count (AFC) was the number of antral follicles detected by ultrasound before oocyte retrieval.

### Statistical Analysis

All results are presented as median (quartile 1 and quartile 3) or number (percentage). Chi-squared test was applied for the comparison of MR, FR and TER between BMI, TC, TG, HDL-C and LDL-C groups. Possion log-linear model was applied for number of oocytes retrieved analysis and logistic regression model for clinical pregnancy and live birth analysis. Models were constructed with BMI, TC, TG, HDL-C or LDL-C as independent variable, respectively. Unadjusted odds ratios (OR) (95% confidence intervals, 95% CIs) were calculated by univariate Poisson log-linear models and adjusted OR (95% CIs) by multivariate Poisson log-linear models after adjustment for age, infertility types, infertility duration, FSH, LH, T, ovarian surgery and ovarian disorders other than PCOS. OR (95% CIs) in logistic regression models for CPR and LBR were adjusted for age, infertility types, infertility duration, embryo types, number of embryos transferred, male factor and endometrium thickness, and OR (95% CIs) for CCPR and CLBR were adjusted for age, infertility types, infertility duration, number of embryos transferred per cycle, number of embryo transfer cycles, male factor and endometrium thickness per cycle. Statistical significance was set at a probability (*P*) value < 0.05. Data analysis was conducted using SPSS 22.0.

## Results

In this study, 230 patients did not have transferrable embryos, so embryo transfer was canceled. A total of 344 embryo transfer cycles from 282 patients including 51 with at least 2 transfer cycles, were included to analyze the relationship between lipid metabolism and clinical outcomes.

The analysis of clinical pregnancy rate and live birth rate were based on the clinical outcomes of 344 cycles, and cumulative clinical pregnancy rate and cumulative live birth rate were based on 282 patients.

### Analysis of Relationship Between Lipid Metabolism and Number of Oocytes Retrieved

Baseline characteristics of 586 patients undergoing unstimulated natural oocyte retrieval were shown in Table 1. The median (Q1 and Q3) of the number of oocytes retrieved was 13 (Miller and Auchus, 2011; Shalom-Paz et al., 2011). Univariate Poisson log-linear analysis showed that overweight (24–27.9 kg/m^2^) was significantly associated with decreased number of oocytes retrieved (OR: 0.940, 95%CI: 0.896–0.987, *P* = 0.013). High TG (≥ 1.76 mmol/L) levels (OR: 1.092, 95%CI: 1.046–1.139, *P* < 0.001) and low HDL-C levels (≤1.04 mmol/L) (OR: 1.127, 95%CI: 1.077–1.179, *P* < 0.001) were significantly associated with increased number of oocytes retrieved. After adjusted for age, infertility types, infertility duration, FSH, LH, T, ovarian surgery and ovarian disorders, correlations between the number of oocytes retrieved and overweight (OR: 0.925, 95%CI: 0.876–0.977, *P* = 0.005), high TC (≥5.18 mmol/L) (OR: 1.103, 95%CI: 1.046–1.162, *P* = 0.001), TG (OR: 1.137, 95%CI: 1.083–1.193, *P* < 0.001), LDL-C (≥3.37 mmol/L) levels (OR: 1.162, 95%CI: 1.093–1.235, *P* < 0.001), low HDL-C (OR: 1.103, 95%CI: 1.048–1.160, *P* < 0.001) were all significant (Table 2).

**TABLE 1:** Baseline characteristics, oocyte maturation and embryo development index of patients.

	**Median (Q1, Q3) or *N* (percentage) (*N* = 586)**
Baseline characteristics^*a*^	
Age (years)	29 (27, 31)
Infertility Duration (years)	3 (2, 5)
Infertility types	
Primary infertility	424 (72.4%)
Secondary infertility	162 (27.6%)
BMI (kg/m^2^)	24.8 (22.1, 27.8)
FSH (nmol/L)	5.80 (4.71, 6.88)
LH (nmol/L)	7.52 (4.53, 11.80)
T (nmol/L)	1.29 (0.79, 2.20)
TC (mmol/L)	4.64 (4.10, 5.20)
TG (mmol/L)	1.36 (0.94, 2.05)
HDL-C (mmol/L)	1.21 (1.03, 1.39)
LDL-C (mmol/L)	2.89 (2.42, 3.39)
Previous ovarian surgery	14 (2.4%)
Ovarian disorders other than PCOS	18 (3.1%)
AFC	24 (23, 32)
Oocyte retrieval cycle	
Number of oocytes retrieved^*a*^	13 (9, 20)
Number of oocyte frozen cycle	74 (12.6%)
MR^*b*^	50.2% (3966/7899)
FR^*b*^	26.8% (2116/7899)
TER^*b*^	12.1% (956/7899)

*^*a*^586 patients undergoing natural oocyte retrieval cycles.*

*^*b*^512 patients with mature oocyte fertilized.*

*T, testosterone; TC, total cholesterol; TG, triglycerides; HDL-C, high density lipoprotein cholesterol; LDL-C, low density lipoprotein cholesterol; AFC, antral follicle counts; MR, maturation rate per oocyte; FR, fertilization rate per oocyte; TER, transferrable embryo rate.*

**TABLE 2:** Poisson log-linear models of BMI, TC, TG, HDL-C, and LDL-C for the number of oocytes retrieved.

	**Number of oocytes retrieved**	
	**Unadjusted OR (95% CI)^*a*^*P-*value**	**Adjusted OR (95% CI)^*b*^*P-*value**
BMI (kg/m^2^)		
<24	Ref.	Ref.
24–27.9	0.940 (0.896, 0.987) ***P* = 0.013**	**0.925 (0.876, 0.977) *P* = 0.005**
≥28	0.988 (0.938, 1.04) *P* = 0.641	0.977 (0.921, 1.037) *P* = 0.443
TC (mmol/L)		
<5.18	Ref.	Ref.
≥5.18	1.047 (0.999, 1.097) *P* = 0.054	**1.103 (1.046, 1.162) *P* = 0.001**
TG (mmol/L)		
<1.76	Ref.	Ref.
≥1.76	**1.092 (1.046, 1.139) *P* < 0.001**	**1.137 (1.083, 1.193) *P* < 0.001**
HDL-C (mmol/L)		
>1.04	Ref.	Ref.
≤1.04	**1.127 (1.077, 1.179) *P* < 0.001**	**1.103 (1.048, 1.160) *P* < 0.001**
LDL-C (mmol/L)		
<3.37	Ref.	Ref.
≥3.37	1.051 (0.995, 1.111) *P* = 0.077	**1.162 (1.093, 1.235) *P* < 0.001**

*Five models with BMI, TC, TG, HDL, or LDL as independent variables respectively; the bold values mean that the *P* value is less than 0.05.*

*^*a*^Univariate Poisson log-linear models.*

*^*b*^Multivariate Poisson log-linear models. Odds ratio after adjustment for age, infertility types, infertility duration, FSH, LH, T, ovarian surgery and other ovarian disorders.*

*BMI, body mass index; TC, total cholesterol; TG, triglycerides; HDL-C, high density lipoprotein cholesterol; LDL-C, low density lipoprotein cholesterol.*

### Analysis of Relationship Between Lipid Metabolism and Oocyte Maturation Quality and Developmental Potential

As shown in Table 1, 512 patients with oocytes fertilized for embryo frozen or transfer were included in this part. MR, FR, and TER were 50.2, 26.8, and 12.1%, respectively (Table 1). Comparisons among BMI groups showed that BMI was significantly negatively associated with MR (*P* < 0.001), FR (*P* < 0.001) and TER (*P* = 0.002) (Table 3). High TG levels (*P* < 0.001) and low HDL-C levels (*P* = 0.026) were also associated with decreased MR (Table 3). No significant difference was found in MR between TC and LDL-C groups as well as MR, FR, TER between TC, TG, HDL-C, and LDL-C groups.

**TABLE 3:** Oocyte maturation quality and developmental potential by BMI, TC, TG, HDL-C, and LDL-C groups.

		**MR**	**FR**	**TER**
BMI	<24	41.5% (1635/3402)	41.7% (878/3402)	46.3% (439/3402)
(kg/m^2^)	24–27.9	31.2% (1231/2566)	30.8% (649/2566)	27.6% (262/2566)
	≥28	27.3% (1075/1896)	27.4% (577/1896)	26.1% (247/1896)
		*P* < 0.001	***P* < 0.001**	***P* = 0.002**
TC	<5.18	49.9% (2893/5801)	26.5% (1536/5801)	11.9% (691/5801)
(mmol/L)	≥5.18	51.1% (1073/2098)	27.6% (580/2098)	12.6% (265/2098)
		*P* = 0.318	*P* = 0.301	*P* = 0.387
TG	<1.76	51.8% (2506/4837)	26.0% (1260/4837)	11.6% (559/4837)
(mmol/L)	≥1.76	47.7% (1460/3062)	28.0% (856/3062)	13.0% (397/3062)
		***P* < 0.001**	*P* = 0.062	*P* = 0.061
HDL-C	>1.04	48.3% (1104/2288)	26.3% (602/2288)	12.8% (294/2288)
(mmol/L)	≤1.04	51.0% (2862/5611)	27.0% (1514/5611)	11.8% (662/5611)
		***P* = 0.026**	*P* = 0.541	*P* = 0.194
LDL-C	<3.37	49.7% (3279/6593)	26.5% (1749/6593)	12.0% (790/6593)
(mmol/L)	≥3.37	52.6% (687/1306)	28.1% (367/1306)	12.7% (166/1306)
		*P* = 0.058	*P* = 0.241	*P* = 0.461

*Data were shown as rate (number of mature, fertilized oocyte or transferrable embryo/number of oocytes retrieved in subgroup); χ*^2^* test; the bold values mean that the *P* value is less than 0.05.*

*BMI, body mass index; TC, total cholesterol; TG, triglycerides; HDL-C, high density lipoprotein cholesterol; LDL-C, low density lipoprotein cholesterol; MR, maturation rate per oocyte; FR, fertilization rate per oocyte; TER, transferrable embryo rate.*

### Analysis of Relationship Between Lipid Metabolism and Clinical Outcomes

Table 4 showed the baseline characteristics of 282 patients undergoing embryo transfer cycles as well as the characteristics of 344 embryo transfer cycles. 191 patients underwent fresh embryo transfer (55.7%) and 91 had embryos frozen for fertility preservation (44.3%). 112 in 344 cycles achieved clinical pregnancy (32.6%) and 56 achieved live birth (16.3%). After at least one embryo transfer cycle, 105 in 282 patients achieved clinical pregnancy (37.2%) and 56 (19.9%) achieved live birth (Table 4).

**TABLE 4:** Embryo transfer cycle characteristics and clinical outcomes.

	**Median (Q1, Q3) or *N* (percentage)**
Baseline characteristics^*a*^	
Age (years)	30 (27, 32)
Infertility duration (years)	4 (2, 6)
Infertility types	
Primary infertility	207 (72.9%)
Secondary infertility	77 (27.1%)
BMI (kg/m^2^)	24.6 (22.4, 27.7)
TC (mmol/L)	4.68 (4.16, 5.24)
TG (mmol/L)	1.40 (0.98, 2.15)
HDL-C (mmol/L)	1.22 (1.04, 1.40)
LDL-C (mmol/L)	2.88 (2.37, 3.36)
Embryo transfer cycle characteristics^*b*^	
Endometrium thickness	8 (7, 10)
Male factor	127 (37.0%)
Embryo types	
Fresh	191 (55.7%)
Frozen	153 (44.3%)
Number of embryo transferred	2 (1, 2)
Clinical outcomes	
CPR	112 (32.6%)
CCPR	105 (37.2%)
LBR	56 (16.3%)
CLBR	56 (19.9%)
Neonatal outcomes^*c*^	
Gestational age (weeks), mean ± SD	36.7 ± 3.6
Premature birth	28.3% (15/53)
Singleton birth	86.8% (46/53)
Birth weight (kg), mean ± SD	3.27 ± 0.59
Birth length (cm), mean ± SD	50.47 ± 2.78
Congenital anomaly at birth	0

*^*a*^Baseline characteristics of 282 patients.*

*^*b*^Embryo transfer cycle characteristics of 344 embryo transfer cycles.*

*^*c*^Neonatal outcomes of 53 patients.*

*BMI, body mass index; TC, total cholesterol; TG, triglycerides; HDL-C, high density lipoprotein cholesterol; LDL-C, low density lipoprotein cholesterol; CPR, clinical pregnancy rate; CCPR, cumulative pregnancy rate; LBR, live birth rate; CLBR, cumulative live birth rate.*

Neonatal outcomes of 3 patients was lost to follow-up. The mean gestational age was (36.7 ± 3.6) weeks. 15 patients had preterm births with gestation less than 37 weeks (Table 4). The mean birth weight and length of the newborns was (3.27 ± 0.59) kg and 50.47 ± 2.78 cm, respectively (Table 4). All the women delivered healthy neonates with no congenital malformations or abnormal growth (Table 4).

Logistic regression analysis showed statistically significant association between obesity (≥28 kg/m^2^) and decreased live birth rate (OR: 0.270, 95%CI: 0.109–0.668, *P* = 0.005) as well as cumulative live birth rate (OR: 0.273, 95%CI: 0.106–0.704, *P* = 0.007) (Table 5). No significant associations were found in other Logistic regression analysis.

**TABLE 5:** Binary logistic regression analysis of BMI, TC, TG, HDL-C, and LDL-C for clinical outcomes in embryos transfer cycles.

	**Clinical pregnancy^*a*^ (*N* = 344)**	**Cumulative clinical pregnancy^*b*^ (*N* = 282)**	**Live birth^*a*^ (*N* = 344)**	**Cumulative live birth^*b*^ (*N* = 282)**
BMI (kg/m^2^)				
<24	Ref.	Ref.	Ref.	Ref.
24–27.9	0.857 (0.488, 1.504) *P* = 0.590	0.784 (0.432, 1.423) *P* = 0.423	0.590 (0.282, 1.234) *P* = 0.161	0.487 (0.226, 1.048) *P* = 0.066
≥28	0.548 (0.296, 1.011) *P* = 0.054	0.598 (0.306, 1.166) *P* = 0.131	0.270 **(0.109, 0.668) *P* = 0.005**	**0.273 (0.106, 0.704) *P* = 0.007**
TC (mmol/L)				
<5.18	Ref.	Ref.	Ref.	Ref.
≥5.18	0.577 (0.330, 1.010) *P* = 0.054	0.561 (0.308, 1.024) *P* = 0.060	0.537 (0.255, 1.133) *P* = 0.103	0.549 (0.252, 1.198) *P* = 0.132
TG (mmol/L)				
<1.76	Ref.	Ref.	Ref.	Ref.
≥1.76	0.871 (0.533, 1.423) *P* = 0.582	1.032 (0.602, 1.768) *P* = 0.908	0.722 (0.374, 1.396) *P* = 0.334	0.905 (0.453, 1.809) *P* = 0.777
HDL-C (mmol/L)				
>1.04	Ref.	Ref.	Ref.	Ref.
≤1.04	1.315 (0.792, 2.304) *P* = 0.270	1.410 (0.791, 2.515) *P* = 0.244	0.816 (0.392, 1.697) *P* = 0.585	0.776 (0.360, 1.671) *P* = 0.517
LDL-C (mmol/L)				
<3.37	Ref.	Ref.	Ref.	Ref.
≥3.37	0.459 (0.231, 1.062) *P* = 0.071	0.491 (0.221, 1.089) *P* = 0.080	0.382 (0.126, 1.156) *P* = 0.088	0.391 (0.126, 1.211) *P* = 0.104

*Models with BMI, TC, TG, HDL-C, or LDL-C as independent variables, respectively; data were shown as adjusted OR (95%CI) and *P*-value; the bold values mean that the *P* value is less than 0.05.*

*^*a*^Odds ratio after adjustment for age, infertility types, infertility duration, embryo types, number of embryos transferred, male factor and endometrium thickness.*

*^*b*^Odds ratio after adjustment for age, infertility types, infertility duration, number of embryos transferred per cycle, number of embryo transfer cycles, male factor and endometrium thickness per cycle.*

*BMI, body mass index; TC, total cholesterol; TG, triglycerides; HDL, high density lipoprotein cholesterol; LDL, low density lipoprotein cholesterol.*

## Discussion

To our knowledge, this is the first study that focuses on the relationship between basal lipid metabolism and ART outcomes in patients with PCOS undergoing unstimulated natural cycles. The results showed that (i) increased number of oocytes retrieved was significantly associated with high lipid metabolic levels; (ii) decreased oocyte maturation quality and developmental potential were related with overweight and obesity categorized by BMI; (iii) poor live birth outcomes of embryo transfer cycles were associated with obesity.

PCOS is always associated with many metabolic disorders with dyslipidemia included, which is proven to affect ART outcomes (De Sousa and Norman, 2016). Unstimulated natural cycles with IVM is a valuable option for women with PCOS because of the reduced risk of ovarian hyperstimulation syndrome (OHSS) (Thessaloniki ESHRE/ASRM-Sponsored PCOS Consensus Workshop Group, 2008). Without the effects of ovarian induction drugs, folliculogenesis in women undergoing unstimulated natural cycles are commonly affected by the physiological and pathological states like internal endocrine and metabolic environment. Exploring the relationship between basal lipid metabolism and ART outcomes of unstimulated natural cycles is crucial to reveal the direct effect of maternal lipid environment on folliculogenesis and oocyte developmental potential, and meanwhile provide information for clinicians to assess the embryonic and clinical outcomes before the start of unstimulated natural cycles. In our study, height, weight, TC, TG, HDL-C, and LDL-C were tested within 3 months of patients’ oocyte retrieval date to ensure these index able to represent the lipid metabolic status around natural cycle. Though lipid metabolism will change during pregnancy (von Versen-Hoeynck and Powers, 2007), these index still represent the basal lipid metabolic status and their association with ART outcomes is meaningful to predict the therapeutic effects of unstimulated natural cycle for infertile individuals with PCOS.

The results of Poisson log-linear models showed that the increased serum TC, TG, LDL-C, and decreased serum HDL-C were protective factors for the number of oocytes retrieved in women with PCOS undergoing unstimulated natural cycles. Cholesterol and TG are known to be the central precursor of sex hormones and main form of stored energy, respectively (Miller and Auchus, 2011), which are crucial for folliculogenesis and oocyte maturation (Van Blerkom, 2011). HDL and LDL are the major cholesterol and triglyceride carriers between liver and extrahepatic tissues in circulation, and their uptake in follicles is mainly in two ways. After the selective filtration of blood-follicle barrier (only allows molecules less than 500kD to pass through), serum HDL-C are taken by SCARB1 receptors on granulosa cells as the follicular fluid component (Fujimoto et al., 2010). Theca layer is the vascular-rich structure in follicle (Richards et al., 2018), and cholesterol ester in LDL-C and HDL-C can be taken in directly by both SCARB1 and LDLR on theca cells (Rhainds et al., 2003). Previous studies showed that maternal high fat exposure could cause irreversible and long term damages to the oocytes including meiotic chromosome abnormality, mitochondrial dysfunction and elevated levels of oxidative stress (Luzzo et al., 2012; Cardozo et al., 2016; Hou et al., 2016). Because women with severe hyperlipidemia are commonly advised to lose weight and reduce fat before starting ART in our center, biochemical lipid levels in most patients with dyslipidemia were only slightly increased and still close to normal level. Therefore, the results revealed that slightly elevated lipid metabolic levels might be beneficial to follicular activation and folliculogenesis.

Overweight and obesity were closely associated with poor oocyte maturation quality and developmental potential in unstimulated natural cycles. Numerous studies in previous on the relationship between BMI and ART outcomes mainly focused on COH cycles and found that obesity had no effect on or reduce the number of oocytes retrieved, MR and FR in COH cycles (McCormick et al., 2008; Bellver et al., 2010; Luke et al., 2011; Kudesia et al., 2018). A retrospective study focused on hCG-primed IVM outcomes found comparable oocyte quality among PCOS patients with different BMI (Shalom-Paz et al., 2011). However, only the number of oocytes retrieved, fertilized oocytes and good quality embryos per cycle were compared (Shalom-Paz et al., 2011), while the rate comparison of MR, FR which are more objective parameters was not included.

Obesity is commonly associated with increased ovarian inflammation and oxidative stress which can impair oocyte nuclear and cytoplasmic maturation, follicular steroidogenesis and ovulation (Snider and Wood, 2019). Diet-induced obese mice revealed more apoptotic follicles, smaller oocytes and impaired maturation ability (Jungheim et al., 2010) due to increased oocyte aneuploidy (Luzzo et al., 2012) and mitochondrial dysfunction (Grindler and Moley, 2013). This study concerning unstimulated natural cycles confirmed this adverse effect of obesity on oocyte maturation quality from a clinical perspective.

Obese patients’ oocytes were usually exposed to high cholesterol, triglycerides and lipoprotein environment, but our research showed no significant association between biochemical parameters and oocyte and embryo quality. This seemingly contradiction can be explained partially by the measurement method and clinical significance of BMI and serum biochemical parameters. BMI, to some extent, is the result of various metabolic disorders with more significant influence on oocyte maturation and embryo quality compared with a certain metabolite.

Our study showed that poor live birth outcomes of embryo transfer cycles were associated with obesity. Some of the previous studies showed that BMI was an important reproductive potential regulator in COH cycles (Sarais et al., 2016). However, unstimulated natural cycles are largely different from COH cycles for the absence of ovarian induction drugs. Obese patients with PCOS undergoing hCG-primed IVM cycles showed comparable pregnancy rate to those who were normal and overweight (Buyuk et al., 2011), which was similar to the uncorrelation between BMI and clinical pregnancy rate in our study. Gestation is characterized by hyperlipidemia which is the body’s metabolic adaptation to pregnancy to save glucose and energy for the fetus (von Versen-Hoeynck and Powers, 2007), which means that the basal serum lipid biochemical parameters levels before oocyte retrieval of unstimulated natural cycles may be largely different from that during pregnancy. In addition, the perinatal complications and drugs also have an impact on clinical outcomes. Therefore, it remains difficult and needs further researches to predict clinical pregnancy and live birth outcomes simply by BMI, TC, TG, HDL-C and LDL-C before unstimulated natural cycles.

The immature oocytes retrieved in unstimulated natural cycles are commonly pretty limited, thus clinicians will make effort to enlarge the number of oocytes retrieved through preoperative assessments and operative skills in the oocyte retrieval to improve ART outcomes. Our results showed that basal lipid metabolic status measured by BMI and biochemical parameters was closely associated with the number of oocytes retrieved and quality of *in vitro* matured oocytes. Though BMI is a canonical measure of body fatness, it can hardly completely represent body’s lipid metabolic status and reflect the influence of maternal lipid metabolism on folliculogenesis and oocyte quality. The significant association between slightly elevated biochemical levels of lipid metabolism (high TC, TG, LDL, and low HDL) and increased number of oocytes retrieved suggested that the biochemical parameters should be considered meanwhile with BMI. Lean and overweight women with slightly increased serum biochemical parameters of lipid metabolism were more likely to obtain more immature oocytes in oocyte retrieval and the management of body weight and serum lipid was pretty important.

As the important indispensable component of follicular microenvironment, lipid metabolites could be transferred across follicular cells and regulate the process of hormones secretion, folliculogenesis and oocyte meiosis. Follicle fluid is the environment that follicular somatic cells directly contact with, thus the relationship of lipid metabolites in serum and follicular fluid and the impact of them in these two places on folliculogenesis, oocyte maturation *in vivo* in women with PCOS remain to be further explored. Besides, the association of maternal lipid metabolism during pregnancy and clinical outcomes after oocyte retrieval of unstimulated natural cycles should be analyzed in the future.

However, there are some limitation in this study. Insulin resistance is an important characteristic of PCOS and had negatively effect on the number of oocytes retrieved in hCG-primed patients with PCOS undergoing IVM cycles in a (Vlaisavljević et al., 2009) but not in another study (Chang et al., 2013). The correlation analysis of 238 patients with fasting serum glucose and fasting insulin tested in our study showed no significant relationship between homeostatic model assessment of insulin resistance (HOMA-IR) and the number of oocytes retrieved (data were not given). Further studies with complete data on insulin resistance are needed to ensure the reliability of the results. In addition, different operators may have an impact on IVM outcomes. Therefore, oocyte retrieval operation and IVM were conducted by the experienced clinicians or embryologists in our center according to the same standard procedures to avoid this effect as much as possible.

## Conclusion

This study clarifies the relationship between basal lipid metabolism and ART outcomes in women with PCOS undergoing unstimulated natural cycles, which has never been discussed before. And we found that there is a direct effect of maternal lipid environment on oocyte and embryo developmental potential as well as clinical outcomes in women with PCOS. The maternal lipid environment of women with PCOS can affect folliculogenesis and developmental potential of *in vitro* matured oocytes. BMI-measured healthy weight and slightly elevated biochemical levels of lipid metabolism were potential predictive factors for the number of oocytes retrieved and should be considered together to assess the oocyte quality and perform intervention before the start of unstimulated natural cycles in clinical practice. In all, this study is helpful to further clarify the endocrine and metabolic factors that influence the reproductive potential of patients undergoing unstimulated natural cycle, and contribute to the determination of clinical treatment schemes for PCOS patients.

## Data Availability Statement

The original contributions presented in the study are included in the article/supplementary material, further inquiries can be directed to the corresponding author.

## Ethics Statement

The studies involving human participants were reviewed and approved by the institutional review board of Peking University Third Hospital. The Ethics Committee waived the requirement of written informed consent for participation.

## Author Contributions

TL took part in the data collection and analysis and the manuscript writing. DL and JQu contributed to the data collection and research discussion. XS contributed to the gynecological surgery and the post-surgical management of the patients. XYZ coordinated the overall oocyte and embryo experiments. JL, RY, SY, and XZ performed the immature follicle aspiration operation. HW, LY, CM, RL, and JQi were involved in the research design and discussion. JY contributed to the oocyte vitrification and thawing, study conception and design, and revision of the manuscript. All authors contributed to the article and approved the submitted version.

## Conflict of Interest

The authors declare that the research was conducted in the absence of any commercial or financial relationships that could be construed as a potential conflict of interest.

## Publisher’s Note

All claims expressed in this article are solely those of the authors and do not necessarily represent those of their affiliated organizations, or those of the publisher, the editors and the reviewers. Any product that may be evaluated in this article, or claim that may be made by its manufacturer, is not guaranteed or endorsed by the publisher.

## References

[B1] Bailey A. P. Hawkins L. K. Missmer S. A. Correia K. F. Yanushpolsky E. H. (2014). Effect of body mass index on in vitro fertilization outcomes in women with polycystic ovary syndrome. *Am. J. Obstet. Gynecol.* 211 163.E1–163.E6. 10.1016/j.ajog.2014.03.035 24657792

[B2] Balen A. H. Morley L. C. Misso M. Franks S. Legro R. S. Wijeyaratne C. N. (2016). The management of anovulatory infertility in women with polycystic ovary syndrome: an analysis of the evidence to support the development of global WHO guidance. *Hum. Reprod. Update* 22 687–708. 10.1093/humupd/dmw025 27511809

[B3] Bellver J. Ayllón Y. Ferrando M. Melo M. Goyri E. Pellicer A. (2010). Female obesity impairs in vitro fertilization outcome without affecting embryo quality. *Fertil. Steril.* 93 447–454. 10.1016/j.fertnstert.2008.12.032 19171335

[B4] Buyuk E. Seifer D. B. Illions E. Grazi R. V. Lieman H. (2011). Elevated body mass index is associated with lower serum anti-mullerian hormone levels in infertile women with diminished ovarian reserve but not with normal ovarian reserve. *Fertil. Steril.* 95 2364–2368. 10.1016/j.fertnstert.2011.03.081 21529798

[B5] Cardozo E. R. Karmon A. E. Gold J. Petrozza J. C. Styer A. K. (2016). Reproductive outcomes in oocyte donation cycles are associated with donor BMI. *Hum. Reprod.* 31 385–392. 10.1093/humrep/dev298 26677960

[B6] Catalano P. M. Shankar K. (2017). Obesity and pregnancy: mechanisms of short term and long term adverse consequences for mother and child. *BMJ Clin. Res.* 356:j1. of interests and have no conflicts to declare, 10.1136/bmj.j1 PMC6888512 28179267

[B7] Chang E. M. Han J. E. Seok H. H. Lee D. R. Yoon T. K. Lee W. S. (2013). Insulin resistance does not affect early embryo development but lowers implantation rate in in vitro maturation-in vitro fertilization-embryo transfer cycle. *Clin. Endocrinol. (Oxf.)* 79 93–99. 23176069 10.1111/cen.12099

[B8] Chen C. M. (2008). Overview of obesity in Mainland China. *Obes. Rev.* 9(Suppl. 1) 14–21. 18307694 10.1111/j.1467-789X.2007.00433.x

[B9] De Sousa S. M. Norman R. J. (2016). Metabolic syndrome, diet and exercise. *Best Pract. Res. Clin. Obstet. Gynaecol.* 37 140–151. 10.1016/j.bpobgyn.2016.01.006 26972165

[B10] Dhanju C. K. Sangha G. K. Sekhon P. K. (2001). Biochemical status of ovaries after induction of superovulation on different days of estrus cycle in mice. *Indian J. Exp. Biol.* 39 777–780. 12018579

[B11] Fujimoto V. Y. Kane J. P. Ishida B. Y. Bloom M. S. Browne R. W. (2010). High-density lipoprotein metabolism and the human embryo. *Hum. Reprod. Update* 16 20–38. 10.1093/humupd/dmp029 19700490

[B12] Grindler N. M. Moley K. H. (2013). Maternal obesity, infertility and mitochondrial dysfunction: potential mechanisms emerging from mouse model systems. *Mol. Hum. Reprod.* 19 486–494. 10.1093/molehr/gat026 23612738 PMC3712655

[B13] Hou Y. J. Zhu C. C. Duan X. Liu H. L. Wang Q. Sun S. C. (2016). Both diet and gene mutation induced obesity affect oocyte quality in mice. *Sci. Rep.* 6:18858. 10.1038/srep18858 26732298 PMC4702149

[B14] Joint committee issued Chinese guideline for the management of dyslipidemia in adults (2016). 2016 Chinese guideline for the management of dyslipidemia in adults. *Zhonghua Xin Xue Guan Bing Za Zhi* 44 833–853. 10.3760/cma.j.issn.0253-3758.2016.10.005 27903370

[B15] Jungheim E. S. Schoeller E. L. Marquard K. L. Louden E. D. Schaffer J. E. Moley K. H. (2010). Diet-induced obesity model: abnormal oocytes and persistent growth abnormalities in the offspring. *Endocrinology* 151 4039–4046. 20573727 10.1210/en.2010-0098 PMC2940512

[B16] Kudesia R. Wu H. Hunter Cohn K. Tan L. Lee J. A. Copperman A. B. (2018). The effect of female body mass index on in vitro fertilization cycle outcomes: a multi-center analysis. *J. Assist. Reprod. Genet.* 35 2013–2023. 30132171 10.1007/s10815-018-1290-6 PMC6240553

[B17] Luke B. Brown M. B. Missmer S. A. Bukulmez O. Leach R. Stern J. E. (2011). The effect of increasing obesity on the response to and outcome of assisted reproductive technology: a national study. *Fertil. Steril.* 96 820–825. 10.1016/j.fertnstert.2011.07.1100 21821244

[B18] Luzzo K. M. Wang Q. Purcell S. H. Chi M. Jimenez P. T. Grindler N. (2012). High fat diet induced developmental defects in the mouse: oocyte meiotic aneuploidy and fetal growth retardation/brain defects. *PLoS One* 7:e49217. 10.1371/journal.pone.0049217 authors have competing interest to report. This does not alter the authors’ adherence to all the PLoS One policies on sharing data and materials 23152876 PMC3495769

[B19] McCormick B. Thomas M. Maxwell R. Williams D. Aubuchon M. (2008). Effects of polycystic ovarian syndrome on in vitro fertilization–embryo transfer outcomes are influenced by body mass index. *Fertil. Steril.* 90 2304–2309. 10.1016/j.fertnstert.2007.10.077 18191852

[B20] Miller W. L. Auchus R. J. (2011). The molecular biology, biochemistry, and physiology of human steroidogenesis and its disorders. *Endocr. Rev.* 32 81–151. 10.1210/er.2010-0013 21051590 PMC3365799

[B21] Moragianni V. A. Jones S.-M. L. Ryley D. A. (2012). The effect of body mass index on the outcomes of first assisted reproductive technology cycles. *Fertil. Steril.* 98 102–108. 10.1016/j.fertnstert.2012.04.004 22584023

[B22] Qiao J. Feng H. L. (2011). Extra- and intra-ovarian factors in polycystic ovary syndrome: impact on oocyte maturation and embryo developmental competence. *Hum. Reprod. Update* 17 17–33. 10.1093/humupd/dmq032 20639519 PMC3001338

[B23] Racowsky C. Vernon M. Mayer J. Ball G. D. Behr B. Pomeroy K. O. (2010). Standardization of grading embryo morphology. *Fertil. Steril.* 94 1152–1153. 10.1016/j.fertnstert.2010.05.042 20580357

[B24] Rhainds D. Brodeur M. Lapointe J. Charpentier D. Falstrault L. Brissette L. (2003). The role of human and mouse hepatic scavenger receptor class B type I (SR-BI) in the selective uptake of low-density lipoprotein-cholesteryl esters. *Biochemistry* 42 7527–7538. 10.1021/bi026949a 12809509

[B25] Richards J. S. Ren Y. A. Candelaria N. Adams J. E. Rajkovic A. (2018). Ovarian follicular theca cell recruitment, differentiation, and impact on fertility: 2017 Update. *Endocr. Rev.* 39 1–20. 10.1210/er.2017-00164 29028960 PMC5807095

[B26] Rotterdam ESHRE/ASRM-Sponsored PCOS Consensus Workshop Group (2004). Revised 2003 consensus on diagnostic criteria and long-term health risks related to polycystic ovary syndrome. *Fertil. Steril.* 81 19–25. 10.1016/j.fertnstert.2003.10.004 14711538

[B27] Sarais V. Pagliardini L. Rebonato G. Papaleo E. Candiani M. Viganò P. (2016). A comprehensive analysis of body mass index effect on in vitro fertilization outcomes. *Nutrients* 8:109. 10.3390/nu8030109 26907340 PMC4808839

[B28] Shalom-Paz E. Marzal A. Wiser A. Almog B. Reinblatt S. Tulandi T. (2011). Effects of different body mass indices on in vitro maturation in women with polycystic ovaries. *Fertil. Steril.* 96 336–339. 10.1016/j.fertnstert.2011.05.076 21704986

[B29] Snider A. P. Wood J. R. (2019). Obesity induces ovarian inflammation and reduces oocyte quality. *Reproduction* 158 R79–R90. 30999278 10.1530/REP-18-0583

[B30] Song X.-L. Lu C.-L. Zheng X.-Y. Nisenblat V. Zhen X.-M. Yang R. (2020). Enhancing the scope of in vitro maturation for fertility preservation: transvaginal retrieval of immature oocytes during endoscopic gynaecological procedures. *Hum. Reprod. (Oxford, England)* 35 837–846. 10.1093/humrep/dez273 32154563

[B31] Thessaloniki ESHRE/ASRM-Sponsored PCOS Consensus Workshop Group (2008). Consensus on infertility treatment related to polycystic ovary syndrome. *Fertil. Steril.* 89 505–522. 10.1016/j.fertnstert.2007.09.041 18243179

[B32] Van Blerkom J. (2011). Mitochondrial function in the human oocyte and embryo and their role in developmental competence. *Mitochondrion* 11 797–813. 10.1016/j.mito.2010.09.012 20933103

[B33] Vlaisavljević V. Kovac V. Sajko M. C. (2009). Impact of insulin resistance on the developmental potential of immature oocytes retrieved from human chorionic gonadotropin-primed women with polycystic ovary syndrome undergoing in vitro maturation. *Fertil. Steril.* 91 957–959. 18321495 10.1016/j.fertnstert.2007.12.062

[B34] von Versen-Hoeynck F. M. Powers R. W. (2007). Maternal-fetal metabolism in normal pregnancy and preeclampsia. *Front. Biosci.* 12:2457–2470. 10.2741/2247 17127255

[B35] Wang L. Y. Wang N. Le F. Li L. Lou H. Y. Liu X. Z. (2015). Superovulation induced changes of lipid metabolism in ovaries and embryos and its probable mechanism. *PLoS One* 10:e0132638. 10.1371/journal.pone.0132638 26167919 PMC4500408

[B36] Zarezadeh R. Nouri M. Hamdi K. Shaaker M. Mehdizadeh A. Darabia M. (2020). Fatty acids of follicular fluid phospholipids and triglycerides display distinct association with IVF outcomes. *Reprod. Biomed. Online* 42 301–309. 33279420 10.1016/j.rbmo.2020.09.024

[B37] Zheng X. Wang L. Zhen X. Lian Y. Liu P. Qiao J. (2012). Effect of hCG priming on embryonic development of immature oocytes collected from unstimulated women with polycystic ovarian syndrome. *Reprod. Biol. Endocrinol.* 10:40. 10.1186/1477-7827-10-40 22621829 PMC3499152

